# Extramedullary hematopoiesis in ribs and severe pulmonary hypertension disease following intermediate beta-thalassemia: a case report

**DOI:** 10.1186/s13256-023-04257-6

**Published:** 2023-12-09

**Authors:** Ali Hossein Samadi Takaldani, Nima Javanshir, Helia Honardoost, Mohammad Negaresh

**Affiliations:** 1 Department of Internal Medicine (Pulmonology Division), School of Medicine, Emam Khomeini Hospital, Ardabil University of Medical Sciences, Ardabil, Iran; 2 Faculty of Medicine, School of Medicine, Ardabil University of Medical Sciences, Ardabil, Iran; 3 Department of Internal Medicine, School of Medicine, Ardabil University of Medical Sciences, Ardabil, Iran

**Keywords:** Extramedullary hematopoiesis, Intermediate beta thalassemia, Ribs

## Abstract

**Background:**

Thalassemia is a type of congenital hemoglobinopathy that falls into the category of hemolytic anemias. Extramedullary hematopoiesis is a complication of this disease, which is a mechanism to compensate for chronic anemia in these patients, and imaging is the best diagnostic method.

**Case report:**

In this report, a 36-year-old Caucasian female patient with intermediate beta thalassemia is presented who, at the time of referral, complained of exacerbated shortness of breath. Imaging showed diffuse expansion masses with soft tissue components in the ribs of both hemithoraxes, leading to the diagnosis of extramedullary hematopoiesis.

**Conclusion:**

Extramedullary hematopoiesis in the ribs is an uncommon finding in patients with thalassemia and is a sign of the severity of the disease and a poor prognostic factor that might be preventable if blood transfusion begins at younger ages.

## Introduction

Thalassemia is hypochromic microcytic anemia caused by a deficiency or decreased synthesis of the globin chain in hemoglobin [1]. Two types of classification are suggested; in one category, it is divided into alpha and beta thalassemia. The three main types of beta thalassemia are major, intermedia, and minor. Another classification of thalassemia defines it as two categories: transfusion-dependent thalassemia (TDT) and non-transfusion-dependent thalassemia (NTDT) [2]. Depending on the severity of the disease, patients thalassemia may present with only incidental findings like anemia or, in severe cases, with significant clinical manifestations such as growth retardation, recurrent infections, hepatosplenomegaly, and heart failure [3]. In NTDT cases, diagnosis can be made by peripheral blood smear (PBS), hemoglobin electrophoresis, and gene analysis. Treatment mainly includes reducing symptoms with infrequent blood transfusions, increasing fetal hemoglobin, iron chelation therapy, and splenectomy. On the other hand, treatment of TDT requires regular blood transfusions with iron chelation therapies and stem cell transplantation [2]. One of beta thalassemia complications is extramedullary hematopoiesis (EMH), a condition in which the production of blood cells occurs out of the bone marrow. Various organs can be involved in EMH, including the spleen, liver, lymph nodes, thymus, heart, breasts, prostate, kidneys, adrenal glands, pleura, posterior peritoneal tissue, skin, peripheral and cranial nerves, and spinal cord, and, rarely, in the lungs and ribs [4].

Another complication in patients with thalassemia is increased pulmonary artery pressure, which is usually secondary to pulmonary artery changes, including persistent vasoconstriction, vascular regeneration, and changes in the extracellular matrix [5].

## Case presentation

The patient was a 36-year-old Caucasian woman with known intermediate beta thalassemia who presented to the hospital with nausea, vomiting, and exacerbated shortness of breath of grade II on the Modified Medical Research Council (mMRC) scale in the last 3 days, along with a dry cough. In hemoglobin electrophoresis from 5 years ago, she had a fetal hemoglobin (HbF) of 36% and a hemoglobin alpha 2 (HbA2) of 5.5% . She also mentioned a history of splenectomy 4 years ago, hospitalization a month ago with the COVID-19 infection, and receiving blood transfusion weekly from 2 months ago. She took a tablet of hydroxyurea 500 mg twice daily as medication. Her vital signs showed a normal blood pressure (125/80), pulse rate (84 beats/min), respiratory rate (14 breaths/min), and oxygen saturation of 92%. During her physical examination, her lungs were clear, and there was jugular venous distention. Her abdomen had no tenderness or hepatomegaly (14 mm), but a scar from a previous splenectomy was visible.

**Fig. 1:**
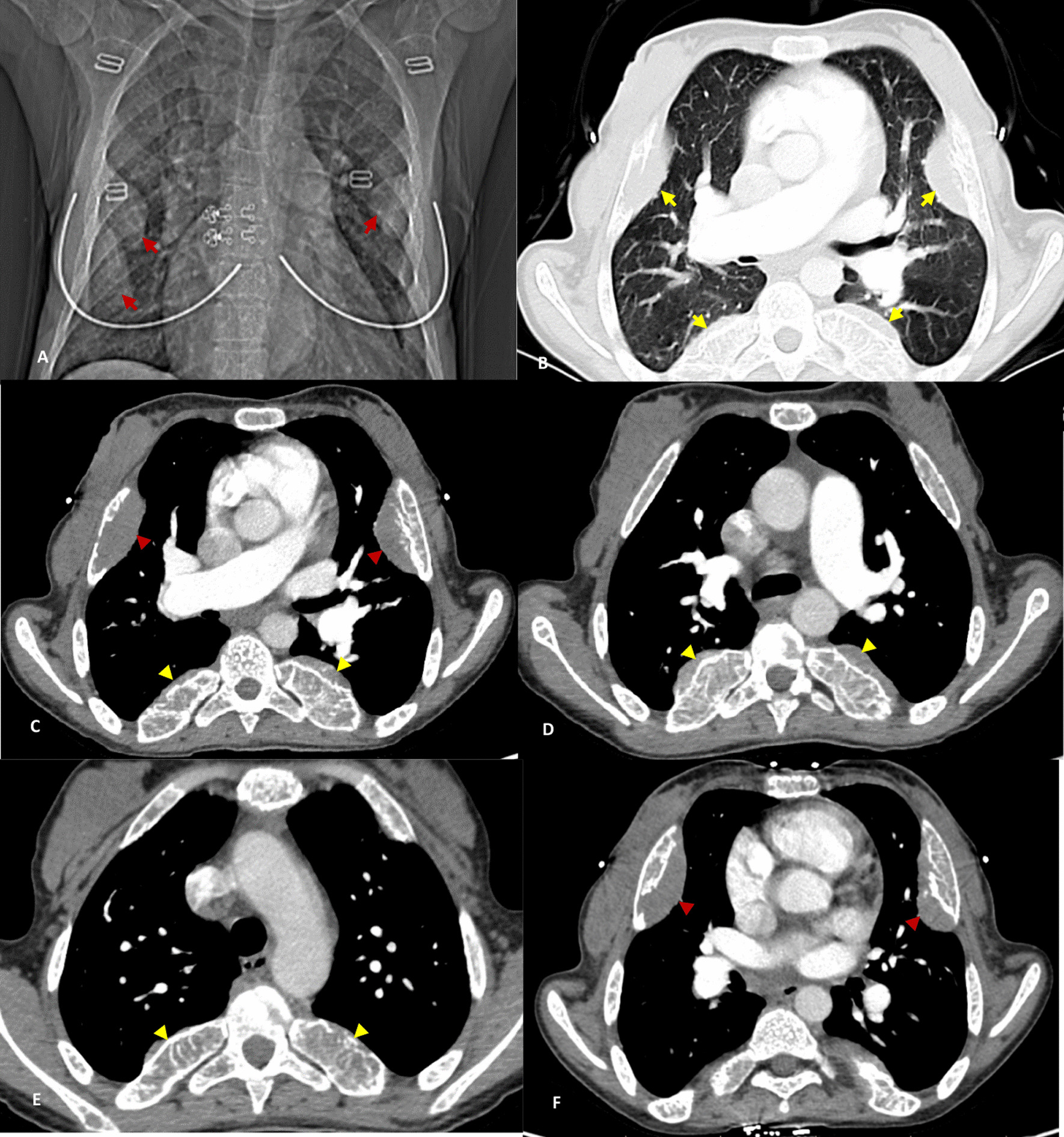
**A** Plain radiography with rib lesions (red arrows). **B** Parenchymal view on computed tomography scan with multiple rib lesions (yellow arrows). **C**–**F** Bilateral rib lesions in lateral (red arrowhead) and posterior sections of ribs (yellow arrowhead)

Table 1 displays the results of the laboratory tests. Chest radiography at the time of admission presented signs of rib enlargement and prominence of the pulmonary artery (Fig. 1A). An echocardiography showed an ejection fraction of 50%, severe pulmonary hypertension (PH) (SPAP = 80 mmHg), D-shape septum during systole due to severe right ventricular pressure overload, severe right ventricular hypertrophy with normal systolic function, and mild mitral regurgitation; the cardiologist suggested a severe increase in pulmonary artery pressure with a noncardiac cause. Due to the echocardiographic findings, a pulmonology consult was requested. The pulmonology consultant ordered a contrast-enhanced chest computed tomography (CT) to rule out a pulmonary embolism. Chest CT imaging showed that the pulmonary trunk was prominent, and its aortopulmonary (AP) diameter was 32 mm. The main arteries of the right and left lungs were also prominent, and no signs of mediastinal adenopathy, lateral fluid, or pulmonary embolism were seen. There was no mass lesion or parenchymal opacity in the parenchyma of the lung field. Rib expansion of both hemithoraxes with soft tissue component in the 4th and 5th ribs of the right and the 4th rib of the left hemithorax was evident. In the left paravertebral of T10 and T9 vertebrae, there was evidence of soft tissue mass with homogeneous density and actual dimensions of 45 × 37 mm (Fig. 1). Findings favor diffuse expansion of the imaged ribs and a left paravertebral soft tissue mass suggestive of EMH as the primary differential diagnosis. Her treatment began with blood transfusions and conservative treatments.

**Table 1:** Results of laboratory tests

	Reference value	Admission	Discharge
WBC (CU/mm)	4000–10000	9700	6900
RBC (10^6^/mm^3^)	4.2–5.4	3.09	3.40
HCT (%)	36–46	24.2	28.1
Hb (g/dl)	12–16	6.6	8.5
MCV (fL)	80–100	93	91
Platelets (10^6^/ml)	150–450	513	220
PTT (seconds)	30–35	30	30
INR (index)	1–1.4	1.0	1.0
Urea (mg/dl)	15–45	27	25
Cr (mg/dl)	0.5–1.4	0.5	0.4
AST (IU/L)	5–40	46	48
ALT (IU/L)	5–40	39	20
ALK. P (IU/L)	64–306	335	249
ESR (mm/hour)	< 23	23	18
CRP	Negative	Negative	Negative
PCR COVID-19	Negative	Positive	–

*WBC* white blood cells, *RBC* red blood cells, *HCT* hematocrit, *Hb* hemoglobin, *MCV* mean corpuscular volume, *Cr* creatinine, *CRP* C-reactive protein, *ESR* erythrocyte sedimentation rate, *INR* international normalized ratio, *PTT* partial thromboplastin time, *AST* aspartate aminotransferase, *ALT* alanine aminotransferase, *ALP* alkaline phosphatase, *PCR* polymerase chain reaction

The next day, her COVID-19 polymerase chain reaction PCR test came back positive. As a result, she was moved to the infectious disease ward for patients with COVID-19. After 2 days, she was discharged with her personal consent and began receiving COVID-19 therapy at home.

## Discussion and conclusion

Patients with intermediate beta thalassemia usually have symptoms between carriers and major beta thalassemia. These symptoms include moderate anemia, which in some patients can cause the need for intermittent blood transfusion with a slightly longer interval than in patients with major beta thalassemia. Patients may suffer from complications caused by ineffective erythropoiesis and hemolysis. These complications include peptic ulcers, PH, and pain [6]. This report presents a case of intermediate beta thalassemia with masses in the paravertebral thorax and ribs, which are an uncommon places for EMH.

EMH is the formation and growth of hematopoietic elements outside the bone marrow, secondary to inadequate erythropoiesis, in processes such as myelofibrosis, thalassemia, hereditary spherocytosis, sickle cell anemia, chronic myeloid leukemia, polycythemia vera, myelodysplastic syndrome, Paget’s disease, osteoporosis, and so on [7]. This is a normal process in the fetus but is considered abnormal after birth [8].

When imaging patients with beta thalassemia, abnormalities such as widening in addition to an individual trabeculated pattern due to osteoporosis involving the entire length of the ribs are often observed. A thin and well-defined cortex and cortical erosions with severe margins may be seen in the lower margin of the ribs and are called “rib within the rib.” Another finding that may be seen is lucencies in the medulla that are small (1–2 mm), well-defined, and localized. It is believed that if the hypertransfusion regimen is started early in life, rib changes will be prevented [9]. The same typical rib findings were evident in our patient.

These rib lesions are possibly associated with EMH, and a high blood perfusion of these tissues makes biopsy a dangerous diagnostic modality [4]. In previous case reports, patients with similar lesions had higher morbidity and mortality than other thalassemia patients after diagnosis and appropriate treatment [10]. Although available thalassemia treatments have prolonged these patients’ lives, long-term side effects of this disease have emerged, one of which is bone involvement [11].

**Fig. 2:**
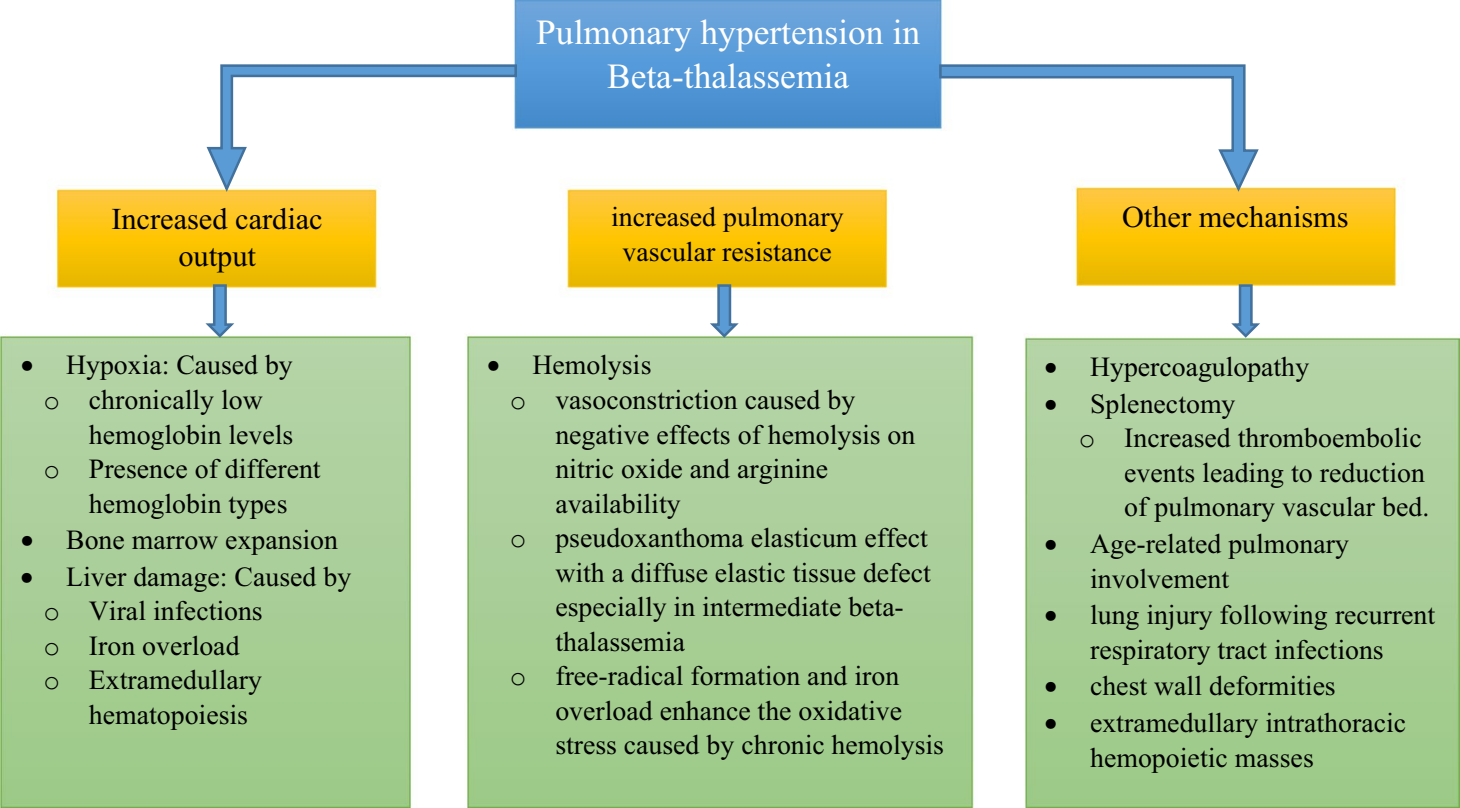
Common mechanisms of pulmonary hypertension in beta thalassemia

PH following thalassemia is classified as the fifth group of the World Health Organization (WHO) classification [12]. Intermediate beta thalassemia-induced PH has a prevalence of 60% and is one of the poor prognostic factors because it has a mortality risk of 22%, 35%, and 40% at 1, 2, and 5 years after definite diagnosis, respectively [13, 14]. The summary of common mechanisms of PH following beta thalassemia is illustrated in Fig. 2 [15].

EMH causing masses in ribs is an uncommon finding in patients with intermediate beta thalassemia. Coexistence of PH and severe EMH is a sign of the severity of the disease and a poor prognostic factor, which might be prevented by screening the patients regularly and starting the blood transfusion treatment at younger ages.

## Data Availability

The datasets used and analyzed during the current study are available from the corresponding author upon reasonable request.

## Abbreviations

mMRC: Modified Medical Research Council
EMH: Extramedullary hematopoiesis
TDT: Transfusion-dependent thalassemia
NTDT: Non-transfusion-dependent thalassemia
PCR: Polymerase chain reaction
PH: Pulmonary hypertension
CT: Computed tomography
SPAP: Systolic pulmonary artery pressure
AP: Aortopulmonary
